# Peptide profile of Parmigiano Reggiano cheese after simulated gastrointestinal digestion: From quality drivers to functional compounds

**DOI:** 10.3389/fmicb.2022.966239

**Published:** 2022-08-23

**Authors:** Vincenzo Castellone, Barbara Prandi, Elena Bancalari, Tullia Tedeschi, Monica Gatti, Benedetta Bottari

**Affiliations:** Department of Food and Drug, University of Parma, Parma, Italy

**Keywords:** long-ripened cheese, Parmigiano Reggiano, bioactive peptides, digestion, bioactivities

## Abstract

Time of ripening has a strong impact on shaping the valuable and recognizable characteristics of long-ripened types of cheese such as Parmigiano Reggiano (PR) due to the interrelationship between microbiota and proteolysis that occurs during ripening. The derived peptide profile is linked to cheese quality and represents the canvas for enzymes upon digestion, which could be responsible for the release of potentially bioactive peptides (BPs). In this study, we aimed at investigating the presence of BP in 72 PR cheese samples of different ripening times, from curd to 24 months of ripening, produced in six different dairies, and following their fate after simulated gastrointestinal digestion. A small number of peptide sequences sharing 100% similarity with known antimicrobial, antioxidant, and ACE-inhibitor sequences were found in PR cheeses, while a higher number of potential BPs were found after their simulated gastrointestinal digestion, in different amounts according to ripening time. Taking advantage of the complex organization of the sampling plan, we were able to follow the fate of peptides considered quality drivers during cheese ripening to their release as functional compounds upon digestion.

## Introduction

Fermented dairy products are consumed from the dawn of time, and nowadays are gaining even more attention due to their potential functional features (van der Reijden et al., 2017; Górska-Warsewicz et al., 2019; Castellone et al., 2021). Scientific evidence is accumulating on the role of long-ripened types of cheese in conferring positive health effects to consumers. As an example, cheese consumption has been associated with a significantly reduced risk of blood hypertension, stroke, and coronary heart disease (Petyaev and Bashmakov, 2012), as well as colorectal cancer (Godos et al., 2020). During the manufacturing of long ripened cheese, many processes take place, from milk gathering to the time when the cheese is finally ready. Among all these processes, ripening is regarded as the most important and despite the static appearance of the cheese wheels in the ripening chamber, it is a deeply active period of radical biochemical changes in the matrix. In particular, proteolysis is recognized as one of the most important events during cheese ripening, and the interrelationship between cheese microbiota and aging results in a specific peptide profile (Bottari et al., 2020), which leads to the valuable and recognizable characteristics of long-ripened types of cheese, and where bioactive peptides (BPs) have frequently been found (Coppola et al., 2000; Ardö et al., 2009; López-Expósito et al., 2017; Di Nunzio et al., 2022). BPs are protein fragments produced from parent proteins, involved in carrying out various physiological functions, such as anti-microbial, antioxidant, antihypertensive, and ACE-inhibitory activities, mainly described for BPs identified in various types of cheese (Sforza et al., 2012; Sultan et al., 2018; Martini et al., 2020; Solieri et al., 2020). Recently, some authors have investigated the presence of BPs in Parmigiano Reggiano (PR) cheese (Basiricò et al., 2015; Martini et al., 2020, 2021; Solieri et al., 2020; Tagliazucchi et al., 2020; Di Nunzio et al., 2022). PR is a protected designation of origin (PDO), raw milk, and hard-cooked cheese, with a minimum ripening time of 12 months (Gatti et al., 2014). PR peptide profile and its evolution during ripening have been described in detail (Sforza et al., 2012; Bottari et al., 2020). Long-ripened kinds of cheese are also known for the presence of Non-Proteolytic Amino Acyl Derivatives (NPADs), which are aminoacyl derivates of non-proteolytic origin synthesized in cheese *de novo* by an enzymatic activity (Bottesini et al., 2014; Zhao et al., 2016; Gazme et al., 2019). These compounds have been reported to exert different bioactivities (Lu et al., 2021). For example, different studies report NPADs being effective in reducing appetite (Yang et al., 2019) and having anti-inflammatory (Xing et al., 2019) and antioxidant effects (Salama et al., 2019), among many others. As they are stable to gastrointestinal digestion, their potential to be absorbed as such and being possibly transported to the body tissues have been suggested (Bottesini et al., 2014). NPADs have been shown to accumulate in PR during ripening, probably due to microbial enzymes (Sforza et al., 2009; Sgarbi et al., 2013).

It is known that gastrointestinal digestion is a key element for determining the biological activities of BPs, which might be degraded or transformed into new sequences released from inactive or less active precursors by pepsin and pancreatic enzymes (Sultan et al., 2018). Lately, the effect of ripening and *in vitro* digestion on the evolution and fate of BPs in PR were studied (Sultan et al., 2018; Martini et al., 2020). In this work, we have exploited the complex sampling that we have already published in a previous work where we described the peptide profile of 72 PR samples as a function of ripening time and microbial dynamics. Leveraging that unique and strictly controlled production and sampling approach, this work was aimed at evaluating the peptide profile of PR at different ripening times and from different dairies after simulated gastrointestinal digestion, particularly focusing on BPs.

## Materials and methods

### Sampling of cheeses

**FIGURE 1:**
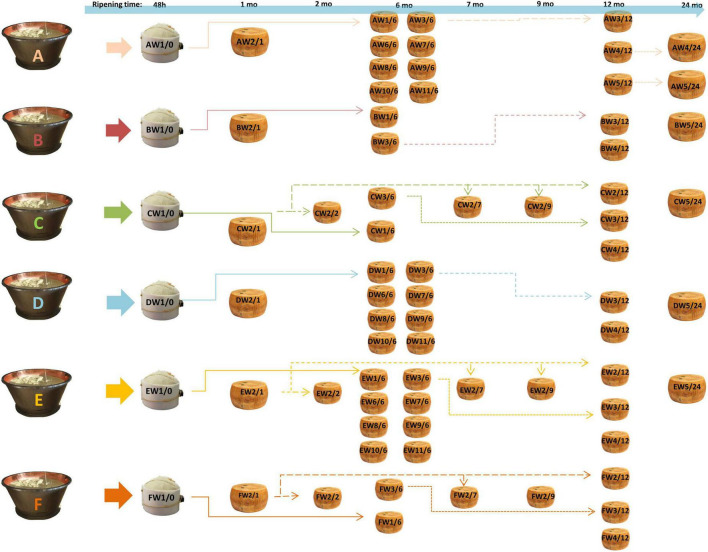
Sampling scheme. For each dairy **(A–F)**, samples were taken from the same cheese-making lot (same wheel, W) at different ripening stages, and from different kinds of cheese-making lots (different wheels) at the same ripening stage. AW1/0 corresponds to dairy A, wheel 1, months of ripening 0, i.e., the curd after 48 h from cheese-making; AW1/6 is the same wheel sampled after 6 months. The same applies to other samples. Samples from the same cheese-making lot are connected by arrows. The time lime of ripening is also indicated at the top of the picture.

The cheese samples were provided by the “Consorzio del Parmigiano-Reggiano” (Reggio Emilia, Italy) and obtained according to Bottari et al. (2020) from six dairies (called A–F) located in the PR PDO production area. For each dairy, samples were taken from the acidified curd (48 h), after brining (1 month of aging) and after 6, 12, and 24 months. For dairies C, E, and F, samples were also taken at 2, 7, and 9 months. Samples were taken for each dairy at different ripening times from the same original wheel (same production batch) and from different wheels with the same ripening times (Figure 1). For each dairy (A–F), the samples were identified with the letter W followed by a number, indicating the sampled wheel, and a slash followed by a second number, indicating the stage of ripening (e.g., AW1/0 corresponds to the dairy A, wheel 1, 0 months of ripening, i.e., the curd 48 h after cheese making). Cheeses were produced according to the EU PDO Regulation established by Article 11 of Regulation (EU) no. 1151/2012 (Gragnani, 2013). The samples were obtained by coring, thus obtaining a cross-section for each wheel. Each section was completely grated and mixed before analysis to have a representative sample of the entire wheel. Aliquots of the samples were kept at –20°C until digestion and subsequent analysis.

### Simulated gastrointestinal digestion

The simulated digestion of the samples was performed according to the INFOGEST protocol (Brodkorb et al., 2019) for gastrointestinal digestion *in vitro* without the addition of gastric lipase. The difficulties in finding the enzyme on the market (despite the high cost), combined with the fact that this study is focused on proteolysis (and not on the lipid fraction) led to this choice. First, the stock solutions were prepared, and then the simulated salivary (SSF), gastric (SGF), and intestinal (SIF) fluids were then prepared by suitably mixing the solutions. The solutions were then brought to 400 ml with demineralized water. In addition to the previous solutions, enzymatic solutions were prepared and suitably dissolved in the corresponding digestive fluid.

After the preparation of the solutions containing digestive fluids and enzymes, 2.5 g of grated PR cheese are weighted, and *in vitro* digestion begins with the oral phase. For the oral phase, we mixed the grated cheese with 1.75 ml of SSF, 250 μL of amylase solution, 12.5 μL of CaCl_2_, and 487.5 μL of demineralized water. The samples were then homogenized for a few seconds with a vortex and incubated for 2 min at 37°C under constant stirring, to simulate chewing. After the oral phase, the gastric phase was continued by adding 3.75 ml of gastric solution, 800 μL of pepsin solution, 2.5 μL of CaCl_2_, 0.3475 ml of demineralized water, and sufficient HCl to the bolus to bring the pH to 3. Then, the gastric phase was carried out in incubation for 2 h at 37°C under constant stirring. The last phase is the intestinal phase, in which 5.5 ml of SIF, 2.5 mL of pancreatin, and 1.25 ml of bile have been added to the chyme, along with 20 μL of CaCl_2_ and enough NaOH to bring the pH to 7. The samples were then incubated at 37°C for an additional 2 h. After incubation, digestion was stopped by bringing the samples to 95°C for 10 min. Samples were then centrifuged at 10,000*g* for 10 min at 4°C and filtered with 0.45 μm sterile syringe filters to remove all the particulates. Finally, to allow for semi-quantification of peptides, samples were spiked with 1 mM of (*L*,*L*)-phenylalanyl-phenylalanine (Phe-Phe) as the internal standard. The samples were then frozen and held at –20°C until ultra-high-performance liquid chromatography-electrospray ionization-tandem mass spectrometry (UHPLC/ESI-MS/MS) analysis. Each sample was extracted and analyzed in triplicate.

### Ultra-high-performance liquid chromatography-electrospray ionization-tandem mass spectrometry analysis

UHPLC/ESI-MS/MS analysis was performed on digested PR samples as described in Prandi et al. (2020). Chromatographic separation was achieved using a reversed-phase column (Aeris Peptide 1.7 μm XB-C18, 150 × 2.10 mm, Phenomenex, Torrance, CA) equipped with a Security Guard ULTRA Cartridge (C18-Peptide, ID 2.1 mm; Phenomenex, Torrance, CA, United States) in a UHPLC system (Dionex Ultimate 3000, Thermo Scientific, Waltham, MA, United States). Eluent A was H_2_O + 0.2% CH_3_CN + 0.1% HCOOH and eluent B was CH_3_CN + 0.2% H_2_O + 0.1% HCOOH. The flow was maintained at 0.2 ml/min and the applied gradient was: 0–7 min, 100% A; 7–50 min, 100% A to 50% A; 50–52.6 min, 50% A; 52.6–53 min, 50% A to 0% A; 53–58.2 min, 0% A; 58.2–59 min, 0% A to 100% A; 59–72 min, 100% A. Total run time: 72 min; column temperature: 35°C; sample temperature: 10°C; injection volume: 2 μL for Full Scan analysis, 4 μL for Product Ion Scan analysis. Detection was achieved using a triple quadrupole TSQ Vantage (Thermo Scientific, Waltham, MA) using the following parameters: positive ion mode, acquisition time: 7–58.2 min (7 min of solvent delay was applied at the beginning of the chromatographic run), acquisition range: 100–1,500 *m*/*z*, micro scans: 1, scan time: 0.50, Q1 PW: 0.70, spray voltage: 3,200 V, capillary temperature: 250°C, vaporizer temperature: 250°C, sheath gas flow: 22 units. The samples were first analyzed in Full Scan mode, then in Product Ion Scan mode. The collision energies (CE) were calculated as CE = 3.314 + 0.034 × m/z (MacLean et al., 2011). The peptides were identified as reported in (Boukid et al., 2019). The peptide sequences were assigned based on the obtained tandem mass spectra. In short, the FindPept software^1^ was used to find the peptide sequences within the target proteins (UniprotKB accessions: P02666, P02662, P02663, P02668, P02754, P00711), whose molecular weight corresponding to the experimental data. Then, the Proteomics Toolkit software^2^ was used to check the correspondence between the theoretical MS/MS fragmentation and the obtained tandem MS spectra.

### Identification of peptides with reported bioactivity in Parmigiano Reggiano samples before and after simulated digestion

After UHPLC/ESI-MS/MS analysis, chromatograms were processed to identify all peptides present in the samples. The peptides from digested samples were semi-quantified against an internal standard (Phe-Phe). After the identification of the peaks and the semi-quantification, the Milk Bioactive Peptide Database (MBPB) was used to identify peptides reported as bioactive in the protein fractions (Nielsen et al., 2017). Peptide sequences with 100% similarity to the recorded BP sequence were considered for the bioactivities. Thirty-four peptide sequences identified in a previous work in the same PR samples before digestion (Bottari et al., 2020), and 105 peptide sequences identified in the present study in digested the PR varieties of cheese were analyzed.

### Statistical analysis

Statistical analyses were carried out using the IBM SPSS Statistics software (version 27.0, Armonk, NY, United States). Bivariate correlation was performed using Pearson’s coefficients, with a two-tailed significance test, and pairwise case exclusion for missing values. Significance was fixed to a *p* < 0.05. The analysis of variance (ANOVA) followed by Tukey’s HSD test was performed to detect statistical differences (*p* ≤ 0.05) among peptides in the samples as a function of ripening time. The SIMCA 16.0.1 (Sartorius Stedim Data Analytics, Göttingen, Germany) software was used to create a principal component analysis (PCA) biplot to get a visual interpretation of the analyzed data.

## Results and discussion

### Peptides resulting from simulated gastrointestinal digestion

The peptide profile of 72 PR cheeses collected from six different dairies (A–F) throughout the PR area, at different ripening times (0, 1, 2, 6, 7, 9, 12, and 24 months) was analyzed after simulated digestion to detect peptides, and in particular, BP, released after the passage in the first part of the GIT. After *in vitro* digestion, 105 different peptides and 13 NPADs were detected. Out of 105 identified peptides, 41 were derived from α-S1-casein and 46 from β-casein. Regarding α-S1-casein, the shortest peptides are composed of 2 amino acids and the longest has 14 amino acids, with an average length of 6 amino acids. On the other hand, the peptides derived from β-casein are composed of peptides among which the shortest are dipeptides, and the longest have 16 amino acids, with an average length of 5 amino acids. Eighteen out of 105 of the detected peptides derived from α-S2-casein and whey proteins.

**FIGURE 2:**
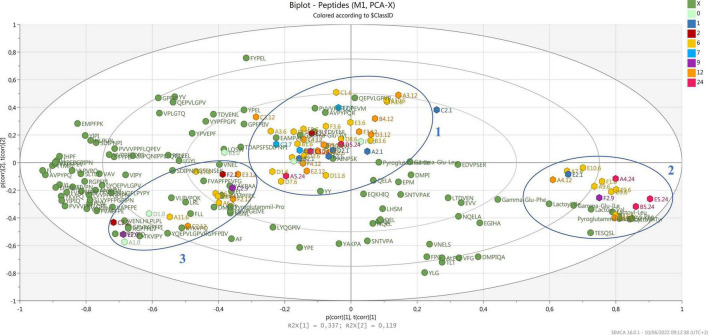
Biplot of samples (hexagons) and all detected peptides (green dots). Samples are colored by ripening time.

Regarding casein digestibility, our results indicate that β-casein is the PR protein most prone to proteolysis, with coverage close to 68%. No peptides derived from regions f(15–43) and f(154–169) were detected, probably indicating that these two regions of the protein are more resistant to proteolysis. A -S1-casein was also highly digestible, with a coverage of 61%; the regions most resistant to proteolytic digestion are those at the N-term of the protein, f(1–16) and f(42–69). Instead, fewer peptides were found from α-S2-casein and κ-casein. This could be due both to a higher resistance of those fractions to proteolysis compared to α-S1-casein and β-casein, and their lower content in PR. Casein coverage after simulated gastrointestinal digestion is shown in Supplementary Figure 1. Clear cleavage sites could not be identified. In fact, pepsin preferentially cleaves in the P1 or P1’ position at Phe, Tyr, Trp, and Leu, but this specificity is lost at pH ≥ 2. Pancreatin is a mixture of different digestive enzymes produced by the exocrine cells of the pancreas. It is a broad-spectrum protease composed of amylase, trypsin, lipase, ribonuclease, and protease. Hence, the complexity of the proteolytic mixture really broadens the possible spectrum of cleavage sites. The principal component analysis (PCA) was performed to investigate possible correlations among peptides detected after digestion and the ripening time of samples (Figure 2).

In the biplot, all the detected peptides and the NPADs (green dots) are reported together with the samples (hexagons) which are differently colored by months of ripening. Samples tend to create a big cluster (cluster 1) in the center of the biplot, where PR from all the dairies of origin and ripening times are present. Most samples (9 out of 16) of 12 months, the minimum ripening time for a PR cheese to be sold, are included in this cluster. A second cluster (cluster 2), characterized by the majority of detected NPAD, can be observed at the bottom right-hand side, where 7 samples of 6 months are present together with 3 samples of 24 months, 2 of 12 months, and 1 of 9 months. Finally, 1 more cluster (cluster 3) can be observed at the bottom left-hand side, grouping samples of different ripening times, and produced in different dairies. These data suggest that after digestion, samples share a common core of a few peptides, while on the other hand, most peptides are widely distributed along the two components, and they do not characterize a specific ripening time. This is not surprising as during ripening the profile of cheese changes a lot because of microbial dynamics (Bottari et al., 2020), with peptides of different sizes and sequences characterizing each time point. Thus, the peptides that after digestion are common to several ripening stages may have been released by both proteins and already hydrolyzed peptides with similar specific cleavage sites (Bottari et al., 2017). Differently from what was previously observed (Bottari et al., 2020), after digestion, samples do not cluster strongly according to the ripening time but are more heterogeneously distributed along the PCOs, as for the 12-month-old PR that is included in all the three clusters. It is known that the most relevant proteolytic transformations in PR cheese occur in the first 12 months (Sforza et al., 2009; Bottari et al., 2020) with a huge peptide evolution, thus peptides at this stage can present many different cleavage sites for digestive enzymes. On the contrary, the majority of NPADs are very strongly related to each other as they have been shown to be perfectly stable to gastrointestinal digestion (Bottesini et al., 2014). These compounds accumulate during ripening, reaching their maximum amount at the end of cheese aging (Sforza et al., 2009). In fact, they were associated with the longer ripened PR varieties of cheese in a former study (Bottari et al., 2020), where all the PR samples from 7 to 24 months clustered with the identified NPAD. However, in this study, where the same samples were analyzed after simulated digestion, only some of the longer ripened cheese samples resulted strongly associated with the majority of the identified NPADs, particularly lactoyl-amino acids. This seems to suggest that the presence of different NPADs is peculiar to different types of cheese.

Interestingly, we observed that samples of 12–24 months of ripening present in cluster 2 have been produced during winter, while those present in cluster 1 were also from summer production (Supplementary Table 1). Seasonal variability in PR production has been previously investigated, both for milk and natural whey starter, although still not exhaustively explained due to the many factors that can affect it (Gatti et al., 2014; Franceschi et al., 2019). In particular, the microbial composition of raw milk has been recently correlated to season (Montel et al., 2014; Garroni et al., 2020). Garroni et al. (2020) showed that lactic acid bacteria were variable from summer to winter, but with a higher abundance of *Lacticaseibacillus* species in winter samples. Considering that this genus, which is coming from raw milk, is dominant in PR cheese up to 20 months of ripening (Neviani et al., 2009) and it has been reported to be responsible for the accumulation of NPADs during PR ripening (Bottari et al., 2020), the correlation found in the present study among PR samples produced in winter and the NPADs in cluster 2 could be due to a higher abundance of *Lacticaseibacillus* species. On the other hand, no specific correlation has been found between ripening and peptides in clusters 1 and 3 despite for those peptides located in the center of cluster 1, namely AINPSK [α-S2-CN f(27-32)], TDVENL [β-CN f(128-133)], γ-Glu-Tyr and QGPIVL [α-S2-CN f(101-106)]. These peptides were in fact more strongly correlated with 12- and 24-months ripened PR that were produced during summer.

As the majority of identified peptides are derived from α-S1-casein and β-casein, two separate PCA were run on data, to evaluate the distribution of the samples and peptides coming from the two native proteins according to ripening time.

**FIGURE 3:**
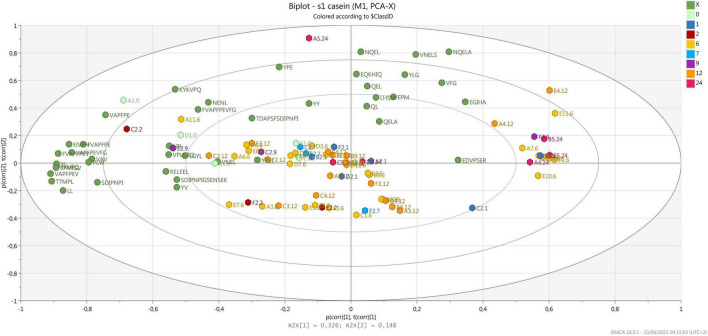
Biplot of samples (hexagons) and peptides derived from α-S1-casein (green dots). Samples are colored by ripening time.

Most samples were grouped according to component 2, correlating with few peptides released after digestion that were derived from α-S1-casein (Figure 3), with no relation to ripening time and area of production. Considering that the same samples clustered according to ripening time when analyzed before digestion (Bottari et al., 2020), the result obtained confirms that *in vitro* digestion greatly influences the peptide profile of PR cheese (Martini et al., 2021). It must be noticed that several samples are grouped together on the right side of the plot, opposite to most peptides derived from α-S1-casein. Most of these peptides contain a proline that is known to increase the resistance to gastro-pancreatic protease action (Tagliazucchi et al., 2016).

**FIGURE 4:**
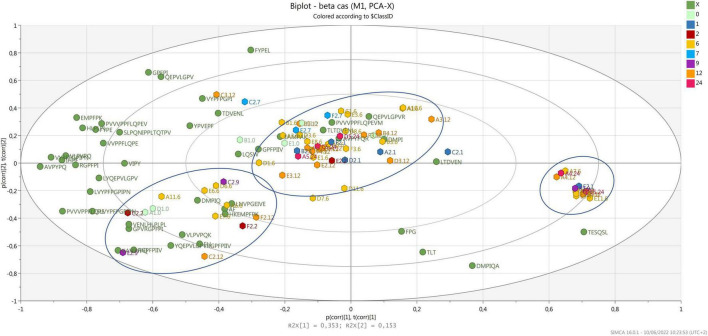
Biplot of samples (hexagons) and peptides d from β-casein (green dots). Samples are colored by ripening time.

Figure 4 shows the biplot with the variance explained for the peptides released after digestion, which are derived from β-casein. In this case, the samples are grouped in three different clusters. Few peptides, characterize one central cluster, where most samples are, meaning that those peptides are released from all cheese samples after digestion, independently from the ripening time and the dairy of origin. Interestingly, most of the 12-month ripened cheese and 2 of the 24-month ripened samples were grouped together in this cluster. A second cluster, characterized by several peptides, is present on the bottom left side of the biplot, where samples from 2 to 12 months of ripening group. Three samples derived from the same cheesemaking (dairy C, same wheel sampled at 2, 9, and 12 months of ripening) are present in this cluster, suggesting that the peptide profile originating from the first biochemical events and evolving during ripening, show a common trend after digestion. Finally, 12 samples mainly from 6 months of ripening group in a third cluster on the right-hand side of the plot, opposite to most peptides derived from β-casein. Also, in this case, these peptides contain a PXP sequence or a proline residue near the carboxylic end, which increases the resistance to gastro-pancreatic proteases action (Tagliazucchi et al., 2016; Martini et al., 2020).

### Peptides with reported bioactivities

The presence of potential BPs was investigated for both undigested (Table 1) and digested cheese samples (Table 2). Analyzing the 34 peptide sequences previously identified in the same PR varieties of cheese before digestion, four potential BPs, and three NPADs were found. One possible BP was derived from α-S1-casein while the other three originated from β-casein. As shown in Table 1, the functionality of BPs present in the PR varieties of cheese of different ripening times, can be multiple, with a single peptide expressing many bioactivities. In fact, 2 of these peptides are reported to exert more than one bioactivity, specifically, the peptide RPKHPIKHQGLPQEVLNENLLRF [α-S1-CN f(1-23)] that was derived from α-S1-casein shows immunomodulatory and antimicrobial activities. However, this potentially bioactive sequence was found only in the peptide profile of PR curd samples. This is not surprising as the length of the peptide makes it easily susceptible to proteolysis in the further ripening stages. On the other hand, the peptides YQEPVLGPVRGPFPIIV [β-CN f(193-209)], DKIHPF [β-CN f(47-52)], and RELEEL [β-CN f(1-6)], respectively, reported as immunomodulator/antithrombin/antimicrobial/ACE-inhibitor, ACE-inhibitor, and antioxidant, were found in almost all the considered ripening stages, albeit in a different amount, with the latter particularly present in 6 months ripened cheeses (data not shown).

**TABLE 1:** List of reported bioactive peptides present in undigested **Parmigiano Reggiano (PR)** cheese samples.

Amino Acids Sequence	Protein	Position	Reported biological effect	References
RPKHPIKHQGLPQEVLNENLLRF	α-S1-casein	1-23	Antimicrobial/Immunomodulatory	Lahov and Regelson, 1996; Birkemo et al., 2009
RELEEL	β-casein	1-6	Antioxidant	Liu et al., 2020
DKIHPF	β-casein	47-52	ACE-inhibitory	Gobbetti et al., 2000
YQEPVLGPVRGPFPIIV	β-casein	193-209	Immunomodulatory/ Antithrombin/Antimicrobial/ACE-inhibitory	Yamamoto et al., 1994; Sandré et al., 2001; Birkemo et al., 2009; Rojas-Ronquillo et al., 2012
Gamma-Glu-Phe			Promotion of cholecystokinin (CCK)/and glucagon-like peptide 1 (GLP-1) secretion	Lu et al., 2021
Gamma-Glu-Leu			DPP-IV Inhibitory, potential functional ingredients in type 2 diabetic diet	Lu et al., 2021
Lactoyl-Tyr			antioxidant	Bottesini et al., 2014

**TABLE 2:** List of the best-known bioactive peptides detected in PR cheese samples after *in vitro* simulated digestion.

Amino Acids Sequence	Protein	Position	Reported biological effect	References
QL	α-S_1_-casein	97–98	DPP-IV Inhibitory/anti-diabetic	Barati et al., 2020
EAMPAK	β-casein	100–105	Antimicrobial	Sedaghati et al., 2016
YY	α-S_1_-casein	165–166	ACE-inhibitory	Fuglsang et al., 2003
YLG	α-S_1_-casein	91–93	Antioxidant/improves cognitive decline	del Mar Contreras et al., 2013; Nagai et al., 2019; Amigo et al., 2020
EL	α-S_1_-casein	39–40, 141–142, 148–149	Antioxidant	Suetsuna et al., 2000
HKEMPFPK	β-casein	106–113	Antimicrobial	Sedaghati et al., 2016
YL	α-S_1_-casein	91–92, 94–95	ACE-inhibitory	Mullally et al., 1996
AVPYPQR	β-casein	177–183	Antioxidant/Antimicrobial/ACE-inhibitory	Rival et al., 2001; Tonolo et al., 2020
VLPVPQK	β-casein	170–176	Antioxidant/Antimicrobial/ACE-inhibitory/Wound healing/Osteoanabolic/anti-apoptotic effect	Shanmugam et al., 2015; Vij et al., 2016; Devi et al., 2020
RELEEL	β-casein	1–6	Antioxidant	Liu et al., 2020
AMKPW	α-S_2_-casein	189–193	ACE-inhibitory	Maeno et al., 1996
EMPFPK	β-casein	108–113	Increase MUC4 expression/Bradykinin-Potentiating/Antimicrobial/ACE-inhibitory	Pihlanto-Leppälä et al., 1998; Perpetuo et al., 2003; Plaisancié et al., 2015; Sedaghati et al., 2016
SDIPNPIGSENSEK	α-S_1_-casein	180–193	Antimicrobial	Hayes et al., 2006
VLPVPQ	β-casein	170–175	Inhibition of cholesterol solubility	Jiang et al., 2020
YPEL	α-S_1_-casein	146–149	Antioxidant	Suetsuna et al., 2000
LNVPGEIVE	β-casein	6–14	ACE-inhibitory	Gobbetti et al., 2000
YPVEPF	β-casein	114–119	Opioid/Increase MUC4 expression/DPP-IV Inhibitory/Antioxidant/Antimicrobial	Jinsmaa and Yoshikawa, 1999; Plaisancié et al., 2015; Nongonierma and Fitzgerald, 2016; Nongonierma et al., 2018
GPFPI	β-casein	203–207	Cathepsin B Inhibitory	Nakagomi et al., 2002
VYPFPGPIPN	β-casein	59–68	Antioxidant	Eisele et al., 2013
VYPFPGPI	β-casein	59–66	prolyl endopeptidase-inhibitory	Asano et al., 1992
YQEPVLGPVRGPFPIIV	β-casein	193–209	immunomodulatory, antithrombin, Antimicrobial, ACE-inhibitory, Immunomodulatory	Yamamoto et al., 1994; Birkemo et al., 2009
Gamma-Glu-Tyr			DPP-IV Inhibitory, potential functional ingredients in type 2 diabetic diet	Lu et al., 2021
Gamma-Glu-Phe			Promotion of cholecystokinin (CCK)/and glucagon-like peptide 1 (GLP-1) secretion	Lu et al., 2021
Gamma-Glu-Leu			DPP-IV Inhibitory, potential functional ingredients in type 2 diabetic diet	Lu et al., 2021

Regarding the analysis of potential BP after digestion, 21 potential BPs and 3 NPADs were detected. Out of these 21 potential BPs, 13 were derived from β-casein, 7 from α-S1-casein, and 1 from α-S2-casein (Table 2). Some of them, specifically 7 out of 20, seem to exert more than one functionality. All the NPADs show more than 1 functionality. Among the range of bioactivities reported for bioactive peptides, one of the most studied features is the ability to inhibit angiotensin-converting enzyme (ACE), preventing a sharp rise in blood pressure, and limiting the risk of heart failure and stroke. Eight BPs {YY [α-S1-CN f(165-166)], YL, AVPYPQR [β-CN f(177-183)], VLPVPQK [β-CN f(170-176)], AMKPW [α-S2-CN f(189-193)], EMPFPK [β-CN f(108-113)], LNVPGEIVE [β-CN f(6-14)], and YQEPVLGPVRGPFPIIV [β-CN f(193-209)]} known for their ACE inhibitory effect (Minervini et al., 2003; Daliri et al., 2018) were detected in the analyzed cheese samples after digestion. One of the BPs {EMPFPK [β-CN f(108-113)]} detected possesses a potential bradykinin-enhancing effect, which in combination with the ACE-inhibitory effect helps to maintain a regular blood pressure level (Castellone et al., 2021). One peptide (QL) is reported to exert antidiabetic effects (Barati et al., 2020). Other known bioactivities attributed to BPs found in the samples include opioid effect and delayed cognitive decline along with inhibition of prolyl-endopeptidase and cathepsin B. These latter effects, combined, could be useful in delaying the progression of tumors (Lee and Lee, 2000; Perez et al., 2020) and fighting the onset and development of Parkinson’s disease (Kalinina et al., 2019). A potential beneficial effect of cheese consumption on cardiovascular health related to the presence of potential BP, or their release after digestion, had already been hypothesized by other authors (Petyaev and Bashmakov, 2012; Hjerpsted and Tholstrup, 2016; Sultan et al., 2018) and for PR (Basiricò et al., 2015; Martini et al., 2020). However, further studies to establish whether the absorption of these peptides in the body is sufficient to develop the bioactive effect are needed (Hjerpsted and Tholstrup, 2016; Santiago-López et al., 2018).

In our experiments, only four potentially BPs and three NPADs were detected in undigested cheese samples, while among the 105 different peptides revealed by the analysis of digested cheese samples, 21 peptides and 3 NPADs showed at least one bioactivity as reported by the Milk Bioactive Peptides Database (MBDP). This is in agreement with the literature that reports a higher number of BP released after digestion than undigested food (Egger and Ménard, 2017; Giromini et al., 2019; Fernández-Tomé and Hernández-Ledesma, 2020; Di Nunzio et al., 2022). Digestion is a key step to freeing BPs from their cryptic form and increasing the probability of having a positive effect exerted by the food components. Only one BP sequence, namely YQEPVLGPVRGPFPIIV [β-CN f(193-209)], was found both before and after digestion meaning that neither digestive enzymes nor gastric acids can hydrolyze it. Indeed, this multifunctional peptide is known to be resistant to digestion, due to a particular conformation (Rojas-Ronquillo et al., 2012). Other BP found in undigested PR cheese does not resist simulated digestion, although some bioactive fragments found in digested cheese may result from larger bioactive sequences found in undigested PR cheese (Liu and Pischetsrieder, 2017; Martini et al., 2020). For example, the antioxidant peptide “EL” in digested PR cheese may be derived from “RELEEL” [β-CN f(1-6)], a peptide with the same reported bioactivity, present in undigested cheese.

### Semi-quantitative amounts of potentially bioactive peptide in Parmigiano Reggiano cheese

Table 3 shows the semi-quantitative data of peptides known as bioactive released during the digestion of differently ripened PR cheese samples. BPs show different trends: some of them display a constant increasing or decreasing trend according to ripening time, while others increase until reaching a plateau, then start to decrease with aging time. It is also fundamental to consider the effect of digestion in releasing encrypted peptides that otherwise are locked in bigger and not active sequences (Udenigwe et al., 2013).

**TABLE 3:** Relative quantification of potential **bioactive peptides (BPs)** in PR cheese with different ripening times after *in vitro* simulated human digestion.

	QL	EA MP AK	Y Y	YL G	EL	HKE MPFP K	YL	AV PY PQ R	VLPV PQK	AMK PW	RE LE EL	EM PFP K	SDIP NPIG SENS EK	VL PV PQ	YPEL	LNVP GEIV E	YP VE PF	GPFP I	VYPFP GPIPN	VYPF PGPI	YQEPV LGPVR GPFPII V	Gamma- Glu- Tyr	Gamma- Glu- Phe	Gamma- Glu- Leu
A1.0	0,59	0,05	0,64	2	0,08	7,25	4,48	0,37	5,72	12,33	0	5,61	0,84	10,66	0,93	0,87	6,43	4,05	15,19	0,69	0,7	0,00	0,00	0,00
A2.1	0,19	0,04	0,41	0,78	0,04	0	1,91	0,04	0,68	4,84	1,53	3,23	0,22	2,86	0,67	0,18	3,13	2,67	6,95	0,52	0,01	0,00	0,00	0,00
A1.6	0,14	0,22	0,88	0,31	0,23	0	1,54	2,92	4,27	4,64	0,44	4,46	1,49	2,11	2,19	0,01	4,26	3,94	9,38	0,86	0,01	0,00	1,90	0,00
A3.6	40,27	0,00	0	0	0	0	2,07	0	0	3,45	1,68	6,51	0,42	8,41	0	0	0	8,45	12,77	0,93	0,03	0,00	0,00	0,12
A6.6	0,71	0,09	0,56	1,74	0,71	0	3,1	0,63	6,21	6,66	0,45	4,54	1,67	11	0	0,24	0	3,58	12,33	0,47	1,41	0,00	0,87	0,00
A7.6	1,23	0,00	0,45	2,18	0,08	0,00	0,00	0,00	0,00	0,00	0,00	0,00	0,03	0,00	0,00	0,03	0,00	0,00	0,00	0,00	0,00	0,00	1,50	0,00
A8.6	0,26	0,18	0,75	1,47	0,42	0	1,77	0	0,6	3,24	0,81	5,5	0,5	7,03	0	0,07	4,9	6,16	9,11	0,64	0,03	0,00	0,84	0,00
A9.6	0,96	0,00	0,45	2,08	0,12	0,00	0,00	0,00	0,00	0,00	0,00	0,00	0,01	0,00	0,00	0,03	0,00	0,00	0,00	0,01	0,00	0,00	1,48	0,00
A10.6	0,13	0,30	0,85	0,29	0,22	0	1,49	2,86	4,9	4,46	0,43	4,97	1,51	2,11	2,24	0,01	0	4,33	9,33	0,86	0,01	0,00	1,80	0,00
A11.6	0,77	0,13	0,73	2,16	040,77	0	3,19	1,15	4,48	7,26	0,84	5,41	1,39	13,29	0	0	0	5,7	16,54	0,78	1,47	0,00	1,00	0,00
A3.12	0,19	0,21	0,03	0,3	0,39	0	1,37	2,53	3,23	3,6	0,43	3,3	1,89	1,39	2,29	0	3,28	2,17	9,28	0,3	0	0,01	0,00	0,13
A4.12	1,34	0,00	0,72	1,39	0,05	0,00	2,96	0,00	0,00	0,00	0,00	0,00	0,00	0,00	0,00	0,01	0,00	0,00	0,00	0,00	0,00	0,00	0,00	0,65
A5.12	40,27	0,00	0	1,31	0,66	0	2,35	0,06	1,16	4	0,48	5,58	0,38	7,4	0	0	0	5,81	10,57	0,52	0,04	0,00	0,00	0,23
A4.24	0,00	0,00	0,70	1,42	0,05	0,00	0,00	0,00	0,00	0,67	0,00	0,02	0,02	0,00	0,00	0,02	0,00	0,00	0,00	0,00	0,00	0,00	1,43	0,00
A5.24	0,87	0,11	0,00	0,35	0,27	1,19	1,12	0,67	0,40	0,72	40,73	1,90	0,65	1,00	4,50	1,25	1,74	2,90	0,43	3,90	0,44	0,00	1,37	0,00
B1.0	0,31	0,00	0,8	1,33	0,05	0	3,21	1,36	4,27	10,97	1,07	6,96	1,07	9,67	1,67	0,06	6,82	7,35	9,37	1,21	0,74	0,00	0,00	0,00
B2.1	0,28	0,18	0,5	0,96	0,05	0	2,64	0,09	1,43	6,62	1,78	5,08	0,35	5,8	0,91	0,29	5,08	4,04	11,32	0,61	0,04	0,00	0,00	0,00
B1.6	0,41	0,12	0,97	1,2	0,42	0	1,95	0	0	5,93	1,19	6,45	0,63	5,88	2,26	0,02	5,88	6,34	13,18	1,12	0,01	0,00	0,00	0,00
B3.6	0,17	0,04	0,52	0,89	0,14	0	1,9	0	0,56	2,99	1,13	4,25	0,19	5,14	0,97	0,08	3,78	4,13	5,36	0,42	0,02	0,00	1,17	0,00
B4.12	0,12	0,00	0	0	0	0	0,61	0,05	0,4	2,54	0,51	5,01	0,12	5,2	1,01	0,02	4,31	4,91	5,81	0,41	0,02	0,00	0,00	0,14
B9.12	0,28	0,04	0,82	1,24	0,64	0	1,79	0	0	2,9	0,64	7,94	0,12	7,22	1,62	0,02	6,03	7,74	8,47	0,81	0,01	0,00	1,32	0,00
B5.24	0,97	0,00	0,58	1,72	0,09	0,19	0,00	0,00	0,00	0,00	0,00	0,00	0,00	0,00	0,00	0,03	0,00	0,00	0,00	0,00	0,00	0,00	1,65	0,00
C1.0	0,12	0,00	0,7	0,92	0,04	0	2,21	0	0	6,77	3,63	6,43	0,12	5,44	0	0,04	5,73	6,04	11,39	1,12	0,02	0,00	0,00	0,00
C2.1	0	0,36	0,09	0,04	0,01	4,5	1,02	5,28	7,31	7,79	0,19	4,79	1,7	0,33	0,66	0	0	0,16	0,71	0,02	0,5	0,02	0,24	0,00
C2.2	0,47	0,00	0,55	1,7	0,06	8,44	3,75	0,37	5,23	18,89	3,84	6,89	1,29	8,34	0	0	0	4,21	21,28	0,53	0,85	0,01	0,00	0,02
C1.6	0,22	0,00	0	0	0	0	1,85	3,07	6,04	5,04	0,85	6,42	1,46	3,51	2,6	0,01	0	5,31	12,43	0,99	0,01	0,01	0,00	0,13
C3.6	0	0,00	0,75	1,21	0,45	0	1,98	0,59	3,26	6,22	1,23	6,87	0,86	8,83	0	0	0	5,95	9,15	0,9	0,48	0,00	0,00	0,00
C2.7	0,37	0,05	0,83	1,58	1,03	0	1,62	0	0	3	0,88	10,58	0,08	9,34	2,06	0,02	7,58	10,91	9,43	1,34	0,02	0,00	1,29	0,00
C2.9	0,29	0,08	0,53	1,65	0,58	0	2,95	0,21	5,12	6,71	1,09	6,64	0,87	6,62	0,94	0,15	7,1	4,64	12,37	0,5	0,17	0,00	1,15	0,00
C2.12	0,55	0,06	0,31	2,61	1,12	0	3,96	0,4	13,32	6,25	0,89	4,39	2,6	8,64	0,91	0,21	5,43	1,33	9,92	0,24	1,43	0,00	0,89	0,00
C3.12	0,29	0,00	0	0	0	0,83	1,83	0	0	2,96	0,75	5,51	0,07	12,09	0	0	8,9	14,03	11,74	1,55	0,03	0,00	0,00	0,41
C4.12	0,38	0,00	0	1,17	1,07	0	2,14	0,88	3,81	5,62	0,5	7,17	0,91	7,49	0	0	0	5,5	8,78	40,73	0,37	0,00	0,00	0,47
C5.24	0,37	0,06	0,73	1,12	1,1	0	2,1	0,83	4,36	3,87	0,35	7,07	0,64	9,06	1,29	0,02	6,15	5,36	6,8	0,7	0,24	0,00	1,08	0,00
D1.0	0,49	0,00	0,54	1,77	0,06	7,57	3,93	0,33	5,38	16,29	0	6,72	1,06	8,55	0,97	0,61	6,59	3,92	15,73	0,5	0,61	0,02	0,00	0,02
D2.1	0,23	0,04	0,51	0,87	0,03	3,03	2,33	0,09	1,67	5,15	0,86	4,69	0,32	4,63	0,89	0,24	3,9	3,58	7,91	0,46	0,03	0,00	0,00	0,00
D1.6	0,36	0,07	0,82	1,56	0,38	4,58	2,32	0	0,44	5,99	1,16	4,91	0,91	4,57	1,35	0,08	4,88	,39	12,07	0,8	0,02	0,00	1,26	0,01
D3.6	0,2	0,08	0,96	1,44	0,22	0	2,03	0	0,23	4,83	1,56	6,47	0,28	7,1	1,49	0,05	5,57	7	9,63	0,8	0,04	0,00	1,29	0,00
D6.6	40,27	0,15	0,51	1,5	0,43	0	3,11	0,38	5,65	9,52	1,55	6,42	0,68	5,93	0,92	0,26	0	3,85	16,08	0,37	0,2	0,00	0,00	0,00
D7.6	0,51	0,03	0,35	1,48	0,54	0	1,54	0,35	7,77	6,4	0,65	4,3	0,91	5,25	0,66	0,2	4,13	1,49	7,56	0,2	0,39	0,00	0,96	0,00
D8.6	0,38	0,00	0,67	1,14	0,39	0	2,11	0,07	0	3,2	1,05	4,26	0,08	3,95	1,44	0,05	4,12	3,93	8,95	0,64	0,01	0,01	0,00	0,09
D11.6	0	0,00	0,47	1,42	0,73	0	2,31	0,61	4,06	4,35	0,53	3,43	1,24	6,83	0	0	0	2,94	9,01	0,39	041,01	0,00	0,00	0,22
D9.6	0,00	0,00	0,46	2,11	0,16	0,00	0,00	0,00	0,00	0,00	0,00	0,00	0,00	0,00	0,00	0,03	0,00	0,00	0,00	0,00	0,00	0,00	1,44	0,00
D3.12	0	0,00	0	0	0	0	0	0	2,79	0	1,44	0	0,96	0	0	0	0	5,03	9,23	0,84	0,14	0,00	0,00	0,00
D4.12	0,31	0,00	0,71	1,23	040,77	0	2,17	0,08	1,25	2,65	0,55	6,78	0,35	6,83	1,17	0,06	5,46	5,63	8,06	0,44	0,03	0,01	0,00	0,00
D5.24	0,21	0,12	0,65	1,11	0,58	0	2,14	0	0,79	2,54	0,28	5,03	0,22	7,41	1,19	0,03	5,5	6,2	6,89	0,41	0,03	0,00	1,32	0,00
E1.0	0,45	0,10	0,75	0,97	0,04	0	3	0	0	0	0	4,49	0,19	4,27	1,26	0,2	4,52	3,87	11,8	0,85	0,01	0,00	0,00	0,00
E2.1	0,96	0,00	0,43	1,65	0,13	0,00	0,00	0,00	0,00	0,00	0,00	0,00	0,00	0,00	0,00	0,00	0,02	0,00	0,00	0,00	0,00	0,00	1,45	0,00
E2.2	0,24	0,00	0	0	0	3,57	2,64	0,06	2,73	5,45	0	5,17	0,55	5,11	1,19	0,18	4,88	3,68	7,64	0,47	0,03	0,01	0,00	0,02
E1.6	0,46	0,00	0,02	0	0	3,82	2,72	0,11	3,1	6,4	1,25	4,79	0,45	4,17	1,33	0,1	,77	3,38	9,52	0,49	0,03	0,00	0,00	0,10
E3.6	0,2	0,08	0,91	0,68	0,45	0	1,98	0	2	3,4	0,57	6,45	1	5,94	2,31	0	4,85	7,1	7,76	0,88	0,02	0,00	0,00	0,00
E6.6	0,3	0,15	0,5	1,54	0,42	0	3,35	0,45	4,28	10,54	1,96	5,22	0,58	4,74	0	0,28	0	3,83	17,91	0,57	0,32	0,00	1,20	0,00
E7.6	0,47	0,00	0,24	0	0	7,08	3,55	0,45	8,95	10,47	1,91	6,97	1,88	6,68	0,99	0,19	7,23	2,1	413,92	0,17	0,63	0,00	0,00	0,17
E8.6	0,34	0,00	0	0	0	2,74	2,49	0,03	0	5,04	0,95	5	0,2	4,56	1,54	0,07	4,89	4,26	9,66	0,77	0,01	0,01	0,00	0,12
E9.6	0,00	0,00	0,00	2,23	0,17	0,00	0,00	0,00	0,00	0,00	0,00	0,00	0,00	0,01	0,00	0,03	0,00	0,00	0,00	0,00	0,00	0,00	1,60	0,00
E10.6	0,00	0,00	0,00	1,83	0,00	0,00	0,00	0,00	0,00	0,00	0,00	0,00	0,00	0,00	0,00	0,00	0,00	0,00	0,00	0,00	0,00	0,00	1,10	0,15
E11.6	1,47	0,00	0,41	2,21	0,13	0,00	0,00	0,00	0,00	0,00	0,00	0,00	0,00	0,00	0,00	0,03	0,00	0,00	0,00	0,00	0,00	0,00	1,49	0,00
E2.7	0,36	0,18	0,97	1,49	0,46	0	2,19	0,07	0	43,53	0,83	3,02	0,33	8,81	1,8	0,04	6,3	8,12	10,58	0,9	0,03	0,00	1,56	0,00
E2.9	0,52	0,19	0,46	2,55	1,05	0	3,94	0,27	10,85	9,04	1,35	6,11	2,71	11,14	1,11	0,25	0	2,99	17,24	0,32	1,38	0,00	1,19	0,00
E2.12	0,14	0,14	0,38	1,25	0,63	0	2,35	0,14	3,26	3,64	0,6	4,36	0,55	6,3	0	0,06	0	3,47	7,55	0,29	0,11	0,00	0,00	0,00
E3.12	0,77	0,08	0,61	1,3	0,55	0	1,61	0,29	3,21	8,57	0,69	6,34	3,32	6,4	2,01	0,02	7,18	3,22	9,23	0,5	0,75	0,01	0,89	0,00
E4.12	0,00	0,00	0,61	2,21	0,09	0,00	0,00	0,00	0,00	0,00	0,00	0,00	0,00	0,00	0,00	0,03	0,01	0,00	0,00	0,00	0,00	0,00	0,00	0,95
E5.24	0,00	0,00	0,56	1,98	0,11	0,00	0,00	0,00	0,00	0,00	0,00	0,00	0,00	0,00	0,00	0,03	0,00	0,00	0,00	0,00	0,00	0,00	1,76	0,00
F1.0	0,1	0,06	0,62	0,75	0,03	0	2,39	0	0	4	0,99	3,95	0,1	4,83	0,94	0,09	3,52	4,77	6,34	0,62	0,01	0,00	0,00	0,00
F2.1	0,15	0,19	0,64	1,02	0,04	0	1,91	0	0	4,96	2,22	4,41	0,31	5,79	0	0,1	0	5,21	10,04	0,65	0,02	0,00	0,00	0,00
F2.2	0,75	0,00	0,27	0	0	6,14	3,82	0,7	13,85	5,26	1,08	5,34	40,62	6,33	40,84	0,24	4,66	1,59	8,91	0,25	0,78	0,00	0,00	0,43
F1.6	0,00	0,00	0,00	2,08	0,13	0,00	0,00	0,00	0,00	0,00	0,00	0,00	0,00	0,00	0,00	0,03	0,00	0,00	0,00	0,00	0,00	0,00	1,57	0,22
F3.6	0,22	0,00	0	0	0	0	2,25	0,06	1,25	3,19	1,16	4,52	0,33	6,15	1,04	0,13	,76	4,87	7,67	0,52	0,03	0,00	0,00	0,07
F2.7	0,19	0,00	0	0,76	0	0	1,79	0	2,91	3,77	0,41	6,71	0,86	5,87	0	0,01	0	7,42	11,25	0,96	0,02	0,01	0,00	0,10
F2.9	1,36	0,00	0,57	1,79	0,09	0,00	0,00	0,00	0,00	0,00	0,00	0,00	0,00	0,00	0,00	0,03	0,00	0,00	0,00	0,00	0,00	0,00	1,85	0,00
F2.12	0,64	0,03	0,4	1,76	1,03	6,21	3,82	0,34	12,8	4,67	1,14	5,47	0,6	5,65	0,73	0,22	4,48	1,84	7,65	0,3	0,49	0,00	0,00	0,00
F3.12	0	0,00	0	1,22	0,52	0	1,59	0	0	2,28	40,71	5,27	0,3	6,15	1,46	0,03	4,12	6,55	6,56	0,67	0,02	0,00	0,00	0,15
F4.12	0,28	0,15	0,88	1,28	0,56	0	2,46	0,03	1,15	3,33	0,56	5,64	0,77	7,9	0	0,03	0	6,25	8,53	0,51	0,04	0,00	1,45	0,00

Samples are grouped by dairy of production (letter A to F) and with increasing (top to bottom) ripening time (numbers after dot, with 0 representing the curd after 48h of molding). Data are colored for each peptide or NPAD according to their relative abundance in the samples along ripening. Differences of color should be picked in the column. The darker is the red color and the higher is the abundance of that peptide in the specific sample compared to other samples from the same dairy but differently ripened.

To obtain a statistical value for these observations, semi-quantification data were analyzed by Pearson’s correlation and ANOVA. The results are reported in Table 4. Statistical analysis shows that peptide “EL” and the NPAD Gamma-glu-phe follow an increasing trend in digested samples according to ripening time. On the contrary, “HKEMPFPK” [β-CN f(105-113)], “YL,” “AMKPW” [α-S2-CN f(189-193)], and “VYPFPGPIPN” [β-CN f(59-68)] follow a decreasing trend in longer ripened kinds of cheese after digestion. In fact, within their sequences, there are different cleavage sites of digestive enzymes (e.g., K, Y, M, and W). The ANOVA analysis confirms that the presence of BPs was statistically different among the samples for 9 out of 20 detected potentially as BPs.

**TABLE 4:** Pearson’s correlation and ANOVA on **bioactive peptides (BPs)** according to ripening time.

		QL	EAMPAK	YY	YLG	EL	HKEMPFPK	YL	AVPYPQR	VLPVPQK	AMKPW	RELEEL	EMPFPK	SDIPNPIGSENSEK	VLPVPQ	YPEL	LNVPGEIVE	YPVEPF	GPFPI	VYPFPGPIPN	VYPFPGPI	YQEPVLGPVRGPFPIIV	GammaGluTyr	GammaGluPhe	GammaGluLeu
Pearsons’s correlation	Months	0,037	–0,079	–0,043	0,084	,341**	-,275*	-,307**	–0,110	–0,111	–,401**	-,305**	–0,210	–0,056	–0,137	0,083	–0,015	–0,096	–0,067	–,337**	0,089	–0,090	–0,181	,342**	0,099
Sign. (two tails)		0,756	0,512	0,717	0,484	0,003	0,019	0,009	0,357	0,355	0,000	0,009	0,076	0,642	0,253	0,486	0,900	0,422	0,578	0,004	0,457	0,455	0,129	0,003	0,406
	**ANOVA**	**QL**	**EAMPAK**	**YY**	**YLG**	**EL**	**HKEMPFPK**	**YL**	**AVPYPQR**	**VLPVPQK**	**AMKPW**	**RELEEL**	**EMPFPK**	**SDIPNPIGSENSEK**	**VLPVPQ**	**YPEL**	**LNVPGEIVE**	**YPVEPF**	**GPFPI**	**VYPFPGPIPN**	**VYPFPGPI**	**YQEPVLGPVRGPFPIIV**	**GammaGluTyr**	**GammaGluPhe**	**GammaGluLeu**
	Sign	0,796	0,272	0,409	0,412	0,004**	0,000**	0,012*	0,866	0,166	0,005**	0,241	0,194	0,620	0,194	0,900	0,077	0,213	0,026*	0,035*	0,351	0,505	0,398	0,000**	0,029*

The green color represents an increasing trend of the peptide according to the ripening of digested samples, the red color represents a decreasing trend of the peptide according to the ripening of digested samples; *p< 0.05. ** p ≤ 0.01.

Since PDO regulation allows the PR cheese to be sold only after a minimum of 12 months of ripening, differences in BP presence were then searched between 12- and 24-months ripened cheeses. Results showed higher amounts (*p*-value of 0.03) of potential BPs in 24 months ripened samples compared to 12 months ripened ones. This is in agreement with other authors (Martini et al., 2021) and can be explained by the higher proteolytic activity of lactic acid bacteria exerted in the longer ripened cheeses (Bottari et al., 2020).

### Frequency of identification of peptides with potential bioactivities

**FIGURE 5:**
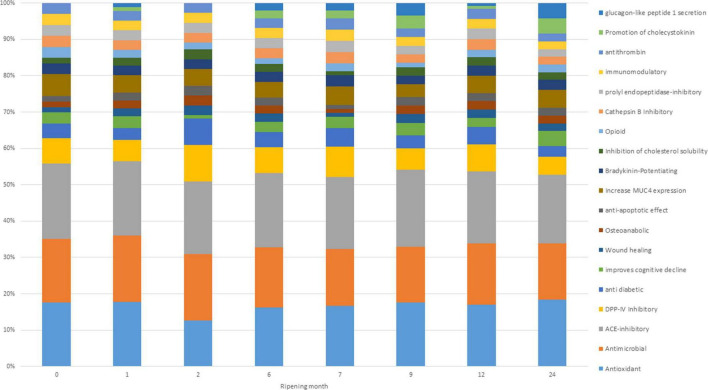
Frequency of identification (in percentage) of bioactivities exerted by detected bioactive peptides (BPs) and non-proteolytic amino acyl derivatives (NPADs) at different ripening times.

Potential bioactivities of digested cheese samples resulting from BP profiles showed small variations based on ripening time (Figure 5). According to the graph, the main bioactivities possibly present at each ripening time are antimicrobial, ACE-inhibitor, and antioxidant. These three bioactivities make up almost 60% of the total bioactivities reported for each single time point. These results agree with Solieri et al. (2020), who reported ACE inhibitory and antimicrobial activities as the most present in PR samples. During digestion, peptides evolve, and their relative quantity increases or decreases, giving rise to changes in the associated bioactivities as well. However, it is not always possible to obtain the maximum beneficial effect from the potential BP present in the PR cheese, since, as indicated by the EU PDO regulation, the PR cheese can only be sold after 12 months of ripening while some bioactivities have the highest peak before reaching the minimum ripening time to be marketed (Figure 5).

The link between dairy intake and health benefits is not entirely clear. Many studies in the scientific literature try to evaluate the production of BP and its absorption in the human body. The big drawback to these experiments is the difficulty in studying the effects *in vivo*. In fact, many of these works concern *in vitro*, *in silico*, or animal approaches (Fu et al., 2016; Cao et al., 2017; Li et al., 2018).

The regular introduction of dairy products into the diet is suggested by many nutritional guidelines for maintaining a healthy diet. The indications also suggest introducing dairy products, especially long-ripened ones, more than once a week. The consumption of long-ripened cheeses has long been debated due to possible negative effects, such as a high content of salt and saturated fats, well known to be linked to an increased risk of heart failure and cardiovascular disease, together with other compounds with possible positive effects. Despite this, diets that involve high consumption of cheese, such as the Mediterranean and French diets, have been shown to reduce the risk of developing non-communicable diseases such as heart attacks and *angina pectoris* (Petyaev and Bashmakov, 2012; Higurashi et al., 2016; Lallès, 2016), suggesting that somehow the positive outcomes of a moderate but constant introduction to the diet of long-ripened cheeses may outweigh the risk posed by the high introduction of salt, saturated fat, and cholesterol (Petyaev and Bashmakov, 2012; Hirahatake et al., 2020; Feeney et al., 2021).

The study of the presence of BP in cheese must take into account not only the production, release, and therefore the presence of peptides in the cheese, but also the mechanisms of release and absorption by the intestinal wall cells (Lorenzo et al., 2018; Sultan et al., 2018). Since many studies in the literature reporting the positive effect of BP are conducted *in vitro*, BP should be tested for its effect *in vivo.*

## Conclusion

The complexity of enzymatic activities that occur during the production of long-ripened cheese has been widely described. This also applies to PR cheese, which despite its restricted geographical area of production is known almost all over the world. Further steps have recently been taken in the literature regarding the understanding of what happens to this treasure of nutrients and possibly bioactive compounds generated after digestion. With this study, conducted on a large number of PR cheese samples with different ripening times and produced by different dairies, we were able to expand what was previously known about the effect of gastrointestinal digestion on the peptide profile of cheese during ripening. The presence of potential BP in samples before and after digestion was also investigated. Samples treated with simulated digestion were characterized by peptide profiles that shared few common peptides, regardless of the ripening time and dairy farm of production. On the other hand, most peptides were released during the digestion of specific samples. The peptide profile is similar after digestion for most PR varieties of cheese at the required minimum ripening time of 12 months. However, differences could be observed among samples of both 12 and 24 months of ripening, possibly linked to the season of production of the cheese. Longer ripened PR varieties of cheese produced during winter showed a stronger relation with NPADs. As the production of these compounds was previously reported to be associated with specific microbial species, such as *Lacticacaseibacillus* spp., further studies are needed to investigate this aspect. As for the potential BP revealed in the samples, they increased with digestion, particularly for long-ripened PR cheeses. Any differences related to the production of dairy could be considered to guide and predict proteolysis from the milk to cheese ready for consumption.

## Data availability statement

The original contributions presented in this study are included in the article/Supplementary material, further inquiries can be directed to the corresponding author.

## Author contributions

VC and BP: analysis. VC, BP, EB, and BB: conceptualization, statistical analysis, and writing. MG and TT: critical review, writing, and editing. All authors contributed to the article and approved the submitted version.

## Footnotes

1
http://web.expasy.org/findpept/

2
http://db.systemsbiology.net/proteomicsToolkit/FragIonServlet.html
