# Supplementary material for: Peptide profile of Parmigiano Reggiano cheese after simulated gastrointestinal digestion: From quality drivers to functional compounds
Source: Front Microbiol. 2022 Aug 23;13:966239. doi: 10.3389/fmicb.2022.966239 (PMC9445588; doi:10.3389/fmicb.2022.966239)
Supplement: Supplementary file 2 [file Table_2.DOCX]

**Supplementary Figure**

**P02662 (α-S1-casein)**

**Coverage: 60.804% (121/199)**

rpkhpikhqg lpqevl**NENL LR**f**FVAPFPE VFG**kek**VNEL**

**S**kdigseste dqamedikqm eaesissse**E IVPN**sv**EQKH**

**IQ**k**EDVPSER YLGYL**eq**LLR L**k**KYKVPQ**l**E IVPN**saeer**L**

**HSM**k**EGIHAQ** qk**EPMIGVNQ ELA**y**FYPEL**f rq**FY**qldayp

sgaw**YYVPLG TQ**y**TDAPSFS DIPNPIGSEN SEKTTMPLW**

**P02663 (α-S2-casein)**

**Coverage: 24.638% (51/207)**

kntmehvsss eesiisqety kqeknm**AINP SK**enlcstfc

k**EVV**rnanee eysigsssee saeva**TEEV**k itvddkhyqk

alneinq**FYQ KFPQYL**q**YLY QGPIVL**npwd qvkrnavpit

ptlnreqlst seenskktvd mestevftkk tklteeeknr

lnflkkisqr yqkf**ALPQYL** ktvyqhqk**AM KPW**iqpk**TKV**

**IPYV**r**YL**

**P02666 (β-casein)**

**Coverage: 67.943% (142/209)**

**RELEELNVPG EIVE**slssse esitrinkki ekfqseeqqq

ted**EL**qdk**IH PF**aqtq**SLVY PFPGPIPNSL PQNIPPLTQT**

**PVVVPPFLQP EVMG**vskvk**E AMAPKHKEMP FPKYPVEPFT**

**ESQSLTLTDV ENLHLPLPLL QSW**mhqphqp lpptvmfppq

svlslsqsk**V LPVPQKAVPY PQRDMPIQAF LLYQEPVLGP**

**VRGPFPIIV**

**P02668 (κ-casein)**

**Coverage: 18.935% (32/169)**

qeqnqeqpir cekderffsd kiak**YIPIQY V**lsrypsygl

n**YY**qqkpval **INNQFLPYPY YAKPAA**vrsp aqilqwqvl**S**

**NTVPAK**scqa qpttmarhph phlsfmaipp kknqdkteip

tintiasgep tstptteave stvatledsp eviesppein

tvqvtstav

**Figure S1.** Casein protein coverage after simulated gastrointestinal digestion. The identified sequences are marked with red capital letters. The signal peptides were removed.
